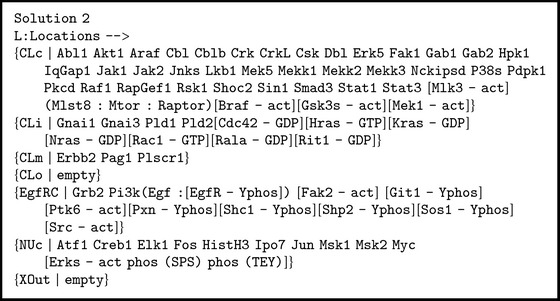# Epidermal Growth Factor Signaling towards Proliferation: Modeling and Logic Inference Using Forward and Backward Search

**DOI:** 10.1155/2017/1809513

**Published:** 2017-01-16

**Authors:** Adrián Riesco, Beatriz Santos-Buitrago, Javier De Las Rivas, Merrill Knapp, Gustavo Santos-García, Carolyn Talcott

**Affiliations:** ^1^Universidad Complutense de Madrid, Madrid, Spain; ^2^Bio and Health Informatics Lab, Seoul National University, Seoul, Republic of Korea; ^3^Cancer Research Center (CSIC/USAL) and IBSAL, Salamanca, Spain; ^4^Biosciences Division, SRI International, Menlo Park, CA, USA; ^5^University of Salamanca, Salamanca, Spain; ^6^Computer Science Laboratory, SRI International, Menlo Park, CA, USA

## Abstract

In biological systems, pathways define complex interaction networks where multiple molecular elements are involved in a series of controlled reactions producing responses to specific biomolecular signals. These biosystems are dynamic and there is a need for mathematical and computational methods able to analyze the symbolic elements and the interactions between them and produce adequate readouts of such systems. In this work, we use rewriting logic to analyze the cellular signaling of epidermal growth factor (EGF) and its cell surface receptor (EGFR) in order to induce cellular proliferation. Signaling is initiated by binding the ligand protein EGF to the membrane-bound receptor EGFR so as to trigger a reactions path which have several linked elements through the cell from the membrane till the nucleus. We present two different types of search for analyzing the EGF/proliferation system with the help of Pathway Logic tool, which provides a knowledge-based development environment to carry out the modeling of the signaling. The first one is a standard (forward) search. The second one is a novel approach based on* narrowing*, which allows us to trace backwards the causes of a given final state. The analysis allows the identification of critical elements that have to be activated to provoke proliferation.

## 1. Symbolic Systems Biology

The technological advances in the analysis of global gene expression and the growth of genomic sequence information have revolutionized research in biology and biomedicine [1, 2]. Investigation of mammalian signaling processes, the molecular pathways by which cells detect, convert, and internally transmit information from their environment to intracellular targets such as the genome, would greatly benefit from the availability of predictive models [3–5].

The goal of our research is to develop abstract qualitative models of metabolic and signaling processes that can be used as the basis of further analyses by powerful tools (e.g., formal verification and model checking with formal methods). We model and analyze the biological processes by which an initial cellular system can lead to the activation of proliferation signaling by using a ligand/receptor pathway. The cells receive external signals by certain biomolecules (ligands) that are able to interact with certain receptors on the cellular surface producing some effects inside the cell [6]. A ligand/receptor system consists of a pathway with multiple elements and known reactions that have been modeled using Maude language by Pathway Logic in a natural, efficient way.

In this work, we use a knowledge base provided by the Pathway Logic system [7]. EGF signaling pathway was selected taking into account its relevance in carcinogenic processes. A novel backward search system has been developed in accordance with the theoretical foundations provided by* narrowing*. Moreover, we present an application of classical forward search with EGF signaling pathway. Authors have published other forward search applications in [8–10].

Various approaches for computational analysis of cellular signaling networks have been proposed to simulate responses to specific stimuli [11, 12]. Simulations using in silico models founded on kinetic measurements of signaling pathways or networks allow us to achieve a detailed understanding of the biochemistry of signal transduction [13]. Ordinary differential equations or stochastic approaches are some of the quantitative methods. The use of differential equations to fit changes in the concentrations from the input to the output is an adequate approach when for a given pathway there are a large amount of quantitative information and a small number of reactions to be modeled [14, 15]. In many cases, however, qualitative approaches (e.g., logic modeling) are more convenient to model complex cell signaling pathways.

Symbolic models are based on formalisms that provide a language to represent the states of a system, mechanisms to model their changes (such as reactions), and tools for analysis based on computational or logical inference. A variety of formalisms have been used to develop symbolic models of biological systems, including Petri nets [16, 17], statecharts [18], live sequence charts [19], ambient/membrane calculi [20], and rule-based systems [21, 22].

In our domain of application, biological interactions can intuitively be specified by rule-based modeling without taking account of the underlying complexity. Rule-based approaches prevent the combinatorial explosion that results from molecular entities existing under multiple conditions. Kappa [23] and BioNetGen [24] are partly similar to Pathway Logic from the rule-based modeling formalisms.

In this paper, Section 1 gives an overview of symbolic systems biology with a rewriting logic and Pathway Logic approach. In Sections 2 and 3, we analyze the role of epidermal growth factor signaling in cancer cell proliferation using narrowing-rewriting techniques. A novel contribution for backward searches in signaling pathways is described. Conclusions are drawn in Section 4.

### 1.1. Cellular Signaling Networks with Pathway Logic Models

Pathway Logic (PL) provides a symbolic systems biology approach to modeling biological processes based on rewriting logic [7, 25, 26]. PL makes it possible to build and analyze models with multiple levels of detail, give information and representations of general rules, define novel data sorts and their properties, and use logical inference for executing queries.

PL primarily allows us to develop abstract qualitative models of signaling and metabolic processes. These models can be used as the basis for analysis by powerful tools to query dynamics on complex biological pathways. A number of recent contributions show their potential for inferring executable models [28] and analyzing the dynamics in different signaling transduction pathways (e.g., nerve growth factor, hepatocyte growth factor, or interleukin-6 signaling pathways) [8–10]. PL also enables developing quantitative and probabilistic models [29].

### 1.2. Rewriting Logic and Maude

Rewriting logic [30, 31] is logic of change that has been successfully applied to represent many different kinds of concurrent systems. A theory in rewriting logic consists of an equational theory, which allows the user to specify sorts, constructors and function symbols (possibly including some equational axioms), and equality between terms. Rewriting logic extends this equational theory by adding the notion of* rewrite rule*, which represents transitions between states.

A simple example of rewriting logic theory specification would be a vending machine that we assume to be composed of a multiset of products (e.g., apples and chocolate) and money (only quarters and dollars are allowed). The sort (i.e., the datatype) required for this theory would be the multiset, whose constructors would be the empty multiset, an apple, a chocolate, a quarter, a dollar, and the union of smaller multisets. Note that the union is commutative (it is unimportant whether we have a quarter and a dollar or a dollar and a quarter) and associative (it is unimportant whether we put together a chocolate with the multiset composed of a quarter and a dollar or we put together the multiset composed of a chocolate and a quarter with a dollar) and has the empty multiset as identity (a multiset does not change by adding the empty multiset). Possible rewrite rules would be the one that transforms a dollar into four quarters, the one that transforms a dollar into a chocolate and one quarter, or the one that transforms two quarters into an apple.

Using the idea of transition between states, it is possible to model biological systems in rewriting logic in a very natural way: while cells are just a set of multisets standing for the different components appearing in a real cell, biochemical reactions are represented by means of rules.

Rewriting logic is efficiently implemented in Maude [32, 33]. In the case of Maude, the underlying equational theory is Membership Equational Logic [34], which in addition to equations allows the user to define membership axioms stating the members of a sort. Maude provides several analysis tools for rewrite theories, including a rewrite computation, breadth-first search, and LTL model checking. Using these features, it is possible to study how our system behaves, to check whether it is possible to reach a certain state from an initial one, and to analyze whether our system verifies some temporal properties. Moreover, since rewriting logic is reflexive [35], a key distinguishing feature of Maude is its metalevel, which allows users to manipulate Maude modules and terms as standard data [36].

## 2. Case Study: EGF Signaling Pathway

We present in this section the case study that will be used to illustrate our technique in Section 3. It is interesting to discuss here the contributions made by these sections: Section 2 describes the pathway of interest and explains how to perform forward analysis, which requires a similar approach to the ones followed by the authors and others when analyzing other pathways. On the other hand, the backward analysis in Section 3 is completely novel; to the best of our knowledge it has never been used in a system following a transitional scheme. Even though the foundations of the analysis are the rules described in Section 2, they are automatically transformed in order to apply the backward search, which in turn is not implemented in the core distribution of Maude and hence requires further modifications to be used. Moreover, this analysis is completely general and can be applied to any other pathway without further modifications.

### 2.1. Epidermal Growth Factor Signaling Pathway

The ErbB family of the receptor tyrosine kinases contains the epidermal growth factor receptor (EGFR) [38, 39]. These receptors couple the binding of the extracellular growth factor ligands to intracellular signaling pathways that control various biologic responses such as proliferation, differentiation, cell motility, and survival [40–45]. Three major steps are involved in the activation of EGFR-dependent intracellular signaling [46]: (a) the binding of a receptor-specific ligand takes place in the extracellular portion of the EGFR or of one of the EGFR-related receptors; (b) the formation of a functionally active EGFR-EGFR dimer or a heterodimer causes the ATP-dependent phosphorylation of specific tyrosine residues in the EGFR intracellular domain; and (c) this phosphorylation triggers a complex program of intracellular signals to the cytoplasm and then to the nucleus.

**Figure 1:**
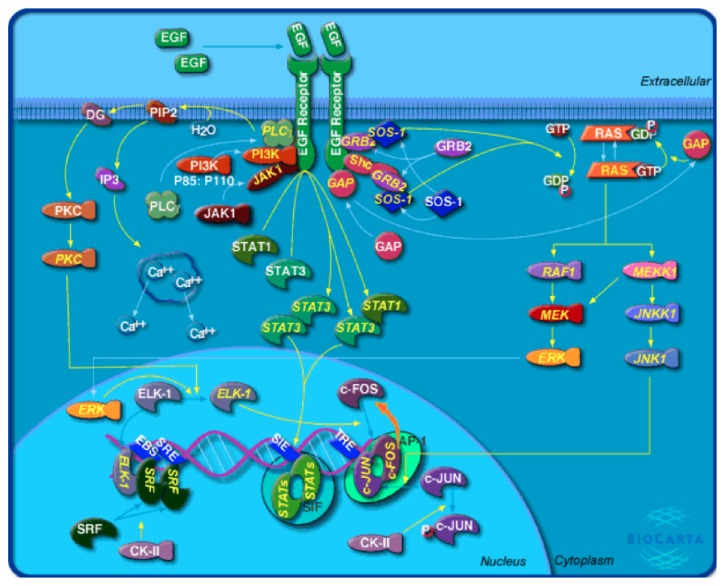
Human EGF signaling pathway (reprinted from Biocarta [27]).

EGFR activates two major intracellular pathways: (i) the RAS-RAF-MEK-MAPK pathway, which controls gene transcription, cell-cycle progression from the G1 phase to the S phase, and cell proliferation, and (ii) the PI3K-Akt pathway, which activates a cascade of antiapoptotic and prosurvival signals: bFGF, basic fibroblast growth factor, HB-EGF, heparin-binding EGF, MAPK, mitogen-activated protein kinase, PI3K, phosphatidylinositol 3,4,5-kinase, TGFa, transforming growth factor alpha, and VEGF, vascular endothelial growth factor (Figure 1).

**Figure 2:**
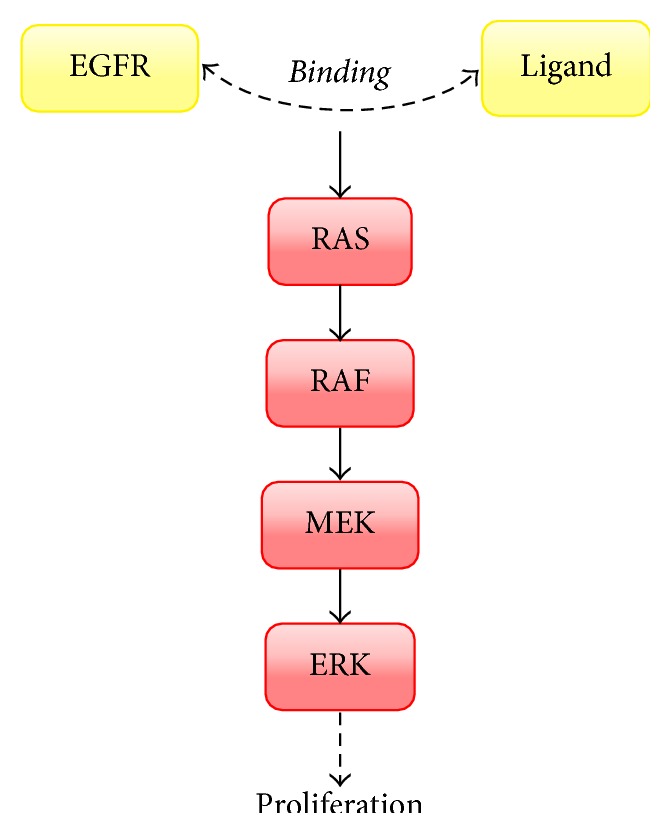
The binding between EGFR and ligand gives rise to intracellular signaling pathways including the RAS/RAF/MEK proliferation pathways [37].

The binding between EGFR and ligand triggers downstream intracellular signaling pathways. Some of them include the PI3K-AKT prosurvival, STAT transcription, and RAS-RAF-MEK proliferation pathways [47]. The RAS-RAF-MEK and PI3K-AKT pathways are mostly activated by the anaplastic lymphoma kinase (ALK) fusion proteins. Cell proliferation, cell motility, and carcinogenesis are driven by the amplification of the EGFR and ALK signaling pathways (Figure 2).

### 2.2. Modeling: Dishes and Rewrite Rules

Some dishes and rules of the STM7 Pathway Logic knowledge base are defined below. A formal knowledge base contains information about the changes that occur in the proteins inside a cell in response to exposure to receptor ligands, chemicals, or various stresses. In our case study, we will focus on models of response to epidermal growth factor (EGF) stimulation. Somatic mutations that lead to EGFR overactivity or upregulation are associated with several types of cancer (e.g., glioblastoma multiforme (GBM), lung cancer, or anal cancers). These mutations involving EGFR lead to its constant activation, which produces uncontrolled cell division. EGFR signal transduction pathways include reactions and, in fact, can induce cellular proliferation activating proteins ERK inside the cells.

An initial state or* dish* (called EgfDish) with several locations and elements is defined:

- The outside (location tag XOut) contains the epidermal growth factor (Egf).
- The EgfRC location contains the epidermal growth factor receptor (EgfR).
- The CLo location, which contains the elements stuck to the outside of the plasma membrane, is empty.
- The membrane (location tag CLm) contains proteins Erbb2, Pag1, and Plscr1.
- The inside of the membrane (location tag CLi) contains several proteins binding to guanosine diphosphate GDP, Cdc42, Hras, Kras, and so forth, and some other proteins, Gnai1, Gnai3, Pld1, and so forth (see the full code shown in Box 1).
- The cytoplasm (location tag CLc) contains enzyme Pi3k and some proteins, Abl1, Akt1, Araf, ArhGap5, and so forth.
- The nucleus (location tag NUc) contains several proteins (Atf1, Creb1, Elk1, etc.).

In Maude syntax, this* dish* (called EgfDish) is expressed by the equation shown in Box 1.

Rewrite rules detail the behavior of cell components depending on biological contexts and modification states. Each rule represents an action in a biological process such as intra/intercellular signaling reactions or metabolic reactions.

#### 2.2.1. Rewrite Rule 001.EgfR.irt.Egf

Pathway Logic contains a set of transition rules, derived from curated experimental findings. They provide an explanation of how a signal propagates in response to an EGF stimulus. Here, we describe rule 001, directly sourced from the literature. Yarden and Sliwkowski [48] determine that when EGF and its relatives bind the ErbB family of receptors, they trigger a network of signaling pathways, culminating in responses ranging from cell division to death and from motility to adhesion.

Our rewrite rule 001 establishes the following*: in the presence of epidermal growth factor* Egf *in the outside of the cell (* XOut *), the receptor* EgfR *is phosphorylated on tyrosine (* [EgfR - Yphos] *) and binds to protein EGF (* Egf) [48, 49]. In Maude syntax, this signaling process is expressed by the rewrite rule shown in Box 2.

#### 2.2.2. Rewrite Rule 188.Shp2.irt.Egf

*When protein EGF (* Egf *) is bound to its receptor EGFR phosphorylated on tyrosine (* [EgfR - Yphos] *) and the cytoplasm contains the tyrosine phosphatase Shp2, then* Shp2 *is phosphorylated on tyrosine and recruited to the* EgfRC *container* [50]. This signaling process is expressed by the Maude rewrite rule shown in Box 3.

#### 2.2.3. Rewrite Rule 529.Hras.irt.Egf

*When protein EGF (* Egf *) is bound to its receptor EGFR phosphorylated on tyrosine (* [EgfR - Yphos] *), the GRB2-associated-binding protein* Gab1 *or* Gab2 *phosphorylated on tyrosine is present (* [gab:GabS - Yphos] *), the Ras guanyl-releasing protein 3* RasGrp3 *or protein* Sos1 *phosphorylated on tyrosine is present,* Pi3k *is present, the protein* Shp2 *phosphorylated on tyrosine is present, and the inside of the membrane (* Cli *) contains* Hras *loaded with guanosine diphosphate* GDP *, then* Hras *switches its load to guanosine 5*′*-triphosphate* GTP [51]. The rewrite rule shown in Box 4 represents this signaling process.

**Figure 3:**
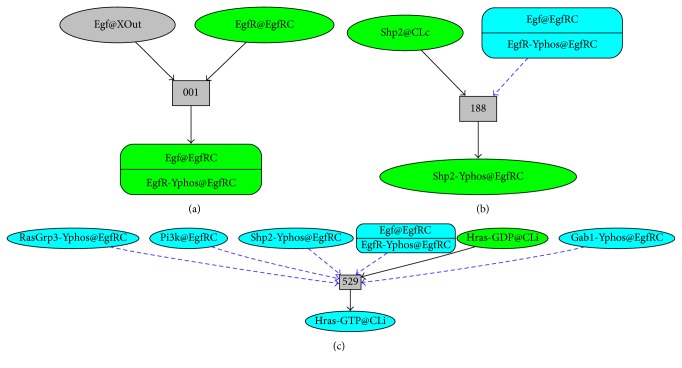
(a) Rule [001.EgfR.irt.Egf] using Pathway Logic Assistant. (b) Rule [188.Shp2.irt.Egf] using PLA. (c) Rule [529.Hras.irt.Egf] using PLA.

Figure 3 shows the aforementioned rules using the Pathway Logic Assistant. An oval represents a component (e.g., gene, protein) participating in a reaction. A rectangle illustrates a reaction rule with a label which represents its shortened identifier in the knowledge base. A solid arrow from an occurrence oval to a rule indicates that the occurrence is a reactant. A dashed arrow indicates that the occurrence is a modifier, enzyme, or control. That is, it is a necessary element for the reaction to take place but is kept unchanged by the reaction. A solid arrow from a rule to an occurrence oval indicates that the occurrence is a product.

### 2.3. Dynamics: Logical Inferences

Our analysis starts with the initial dish state EgfDish defined in Section 2.2 and derived from the knowledge base provided by Pathway Logic. It is a well-known fact that cell proliferation is connected to activation of Erks [52]. We want to find out whether there is a pathway from EgfDish leading to activation of Erks. In this case, one can use the search command with a suitable search pattern and parameters ([n]: the first *n* solutions; =>+: at least one step). The target state is defined by the operator PD, whose argument is a “soup” of locations with their respective contents. A soup is a multiset that can include several elements regardless of their order.

The contents of each location (e.g., EgfRC) are elements and/or variables (e.g., thEgfRC:Things) according to the matching criteria of our search. In the nucleus, a protein prot:BProtein must be activated and can also have a set of other modifiers mod:ModSet. The search condition imposes that the variable prot:BProtein has membership in the sort ErkS (see Box 5).

The solution to this query given by Maude shows the matching in the previous search pattern. While the terms fixed by the search pattern are not shown (e.g., [EgfR - Yphos]: Egf), the variables are presented with their corresponding values. The first solution has the values shown in Box 6.

In this solution, we observe that the variable on-the-fly prot:BProtein matches protein Erks with modifications phos (TEY) and phos (SPS). We find out proteins Braf, Gsk3s, Mlk3, and Mek1 in an activated form in the cytoplasm. We show evidence of a ligand/receptor effect: epidermal growth factor (EGF) binds a specific cell surface receptor (EGFR). Then, we ask Maude for the rule labels which have been applied to reach the final state according to the solution < (see Box 7). Some of these rules are the rewrite rules 001.EgfR.irt.Egf, 188.Shp2.irt.Egf, and 529.Hras.irt.Egf described in Boxes 2, 3, and 4.

This way, Maude allows us to explore the complete search space following a breadth-first strategy until all solutions are found.

## 3. Searching for Causes

In the previous section, we used a standard (forward) search to analyze the set of reachable states from an initial dish; now, we can use* narrowing* to analyze the possible initial states leading to a particular state. That is, in this section, we propose a backward search for analyzing the causes leading to different scenarios. We will first introduce the theoretical notions underlying our scheme, and then we will show how the search works.

Narrowing [53–55] is a generalization of the term rewriting, allowing free variables in terms and replacing pattern matching by unification in order to reduce these terms. It was first used to solve equational unification problems [56] and then generalized to deal with symbolic reachability problems [57]. More formally, the difference between a rewriting step and a narrowing step is that in both cases we use a rewrite rule *l*⇒*r* to rewrite *t* at a position *p* (we express this subterm as *t*|_*p*_), but narrowing unifies the left-hand side *l* and *t*|_*p*_; that is, it uses a substitution *σ* such that *lσ* = _*A*_ *t*|_*p*_ *σ* before actually performing the rewriting step, while in rewriting *t*|_*p*_ must be an instance of *l* (i.e., only matching is required). From an initial term *t* only containing variables (except for the function symbol at the top), we can obtain a substitution *σ*, using this narrowing approach, and generate a term *tσ*, such that *tσ* can be rewritten using the traversed rules to some term *t*′. The current implementation of narrowing in Maude has been applied to symbolic model checking [58] and test-case generation [59], among others.

Recalling our example for the vending machine from the previous section, it is easy to see the difference between standard rewriting and narrowing. Remember that we have a rule in our system that turns two quarters into an apple; in this system, we might wonder whether it is possible to reach a state including an apple starting from a system with two quarters. Using rewriting, we reach a positive answer, as well as starting from a state with three quarters, from a state with one dollar (since we can use the rule to transform it into four quarters), and, in general, from any initial state containing two or more quarters. However, all these initial states must be concrete and contain either quarters, dollars, apples, or chocolates. Applying rewrite to an initial state containing a quarter and a variable standing for a multiset would return a negative answer, because rewriting is not able to assign values to the variable (e.g., a quarter in our case) and hence cannot apply rules. Narrowing, on the other hand, is able to assign values to variables in order to apply rules, so a narrowing search would return a positive result and the required substitution (our variable would be just an apple in 0 steps, a quarter in 1 step, a dollar in 2 steps, etc.).

We can use the rule 01.EgfR.irt.Egf from Section 2 to illustrate the difference between rewriting and narrowing. First, for applying rewriting, we can only start from ground terms (terms without variables), so we could start with the term {XOut ∣ Egf}  {EgfRC ∣ EgfR} and obtain the following result by applying the rule {XOut ∣ none}  {EgfRC ∣ [EgfR - Yphos]: Egf}.

Note that starting with a ground term greatly limits the power of the search, since (i) a slight variation in this term might imply huge differences in the reachable terms and (ii) it limits the causes of the reachable states to that dish, hence ignoring other simpler or more general causes. In order to solve this problem, narrowing is applied to terms with variables that stand for potentially infinite sets of ground terms. In that case, we could start the process with the term {XOut ∣ xout}  {EgfRC ∣ egfrc}. This way, we do not impose any constraint to the initial term. The narrowing process would find that the variable xout must be of the form xout' Egf, while egfrc must be of the form egfrc' EgfR, for xout' and egfrc' fresh variables. This way, we would obtain the term {XOut ∣ xout'} {EgfRC ∣ egfrc' ([EgfR - Yphos]: Egf)}, which is more general than the one above. Since the narrowing process returns the bindings applied to the initial variables, we can use it to check whether a state is reachable from a general term and then obtain the most general initial term required to reach this state, hence allowing us to discard the infinite initial dishes that are a particularization of this solution.

However, due to its computational power, narrowing poses a number of important restrictions. First, it can only be applied to unconditional theories. Secondly, since unification is only available for some particular theories, only some combinations of equational axioms are allowed. Finally, it is required for all the rewrite rules to be defined at the top (the rules must be applied to the complete term and not just to the subterms). Luckily enough, the Pathway Logic specification fulfills the first two constraints, although it fails to fulfill the last one. For this reason, we have developed a way to apply narrowing to any pathway. It is worth discussing the entity and complexity of this extension, whose main points are as follows: (i) it is a metalevel function, which is able to modify the rules in a given module to make them fulfill the constraints above, and (ii) it is Full Maude [32], since narrowing is not implemented in the built-in distribution of Maude but in its Full Maude extension. The former poses theoretical difficulties, since it requires a deep understanding of the structure of Maude modules, terms, and sorts at the metalevel, while the latter poses technical difficulties, since it requires the programmer to deal with the input/output mechanisms and the explicit database used by Full Maude. This extension is available at https://github.com/ariesco/pathway.

For example, we look for the different dishes that can produce the first result for the search in Section 2.3 by using Box 8.

Although the first solution just indicates that we can reach this result by applying no rewrite rules and starting with the same substitution we are looking for, the second solution goes one step backwards and proposes the initial state shown in Box 9.

By looking for further solutions, Maude keeps going backwards until the initial dish is as general as needed by the user. Also note that the initial dish can be further specialized to indicate that we expect some initial components or to focus in a particular element. We consider that this technique, which has been proposed for the first time in this paper, will be very useful for analyzing the causes of many pathways.

## 4. Conclusions

In the last few years, the analysis of biological systems has required an overwhelming and boundless amount of quantitative data with the use of new technologies [60–63]. Obtaining quantitative data in vivo and/or in vitro is often complicated due to ethical aspects or certain limits concerning experimentation. Quantitative methods for modeling and analysis of biochemical networks are infeasible in many cases.

The dynamics of complex biological systems can be explained by developing symbolic methods [64]. Molecular biologists can formalize models to think about signaling pathways and their behavior, allowing them to computationally raise questions about their dynamics and outcomes.

Rewriting logic provides a logical framework that gives us the ability to build and analyze models with multiple levels of detail [32]. Different sorts of elements (proteins, genes, cell locations, etc.) and their properties can be defined. Biological rules can be easily represented. Maude language allows us to perform searches using logical inference.

In this paper, we have described two different searches for studying the epidermal growth factor in the Maude implementation of the Pathway Logic. The first one allows the user to study how different particular dishes can evolve by applying a breadth-first search by rewriting, hence being able to check whether a certain state is reachable. The second search presents a novel approach by applying narrowing instead of rewriting, which allows the user to trace back the possible origins of a given scenario, hence providing a causality study. In order to perform this analysis, we have extended Full Maude, the tool where the narrowing capabilities of Maude are implemented, with a command that preprocesses the Pathway Logic modules in order to fulfill the narrowing requirements. We expect that this novel approach will be useful for analyzing other pathways where the forward approach has already been used.

## Figures and Tables

**Box 1:**
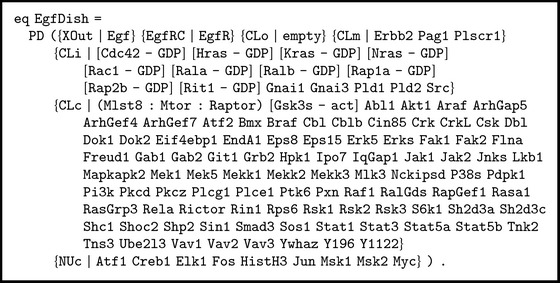


**Box 2:**
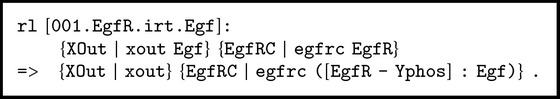


**Box 3:**
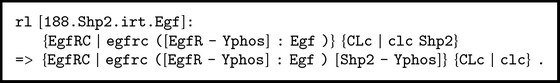


**Box 4:**
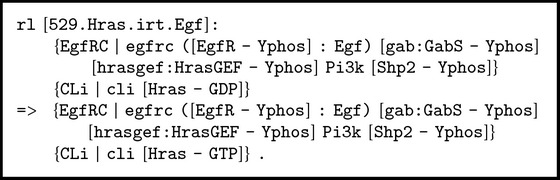


**Box 5:**
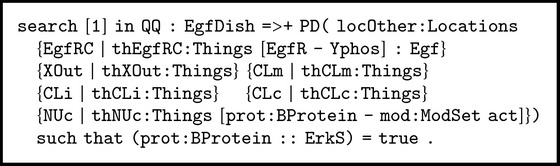


**Box 6:**
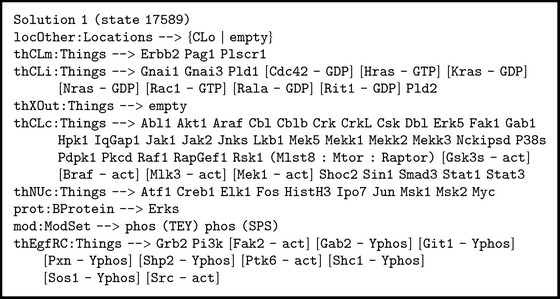


**Box 7:**
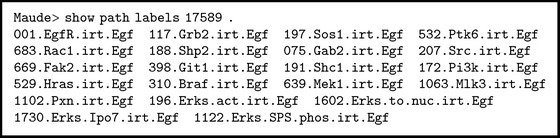


**Box 8:**
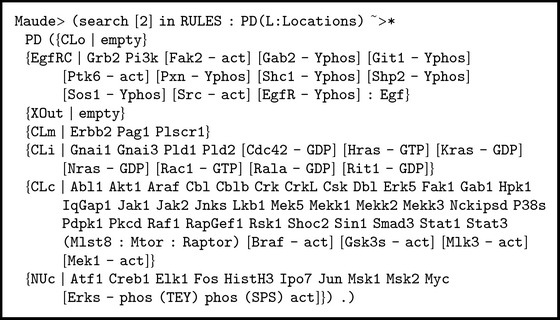


**Box 9:**